# 3D Reactive Oxygen Species Dosimetry in Pleural Photodynamic Therapy: Integration of Macroscopic Kinetic Modeling and Deformable Registration

**DOI:** 10.3390/antiox15050616

**Published:** 2026-05-13

**Authors:** Hongjing Sun, Michele M. Kim, Andreea Dimofte, Sunil Singhal, Keith A. Cengel, Timothy C. Zhu

**Affiliations:** 1 Department of Radiation Oncology, University of Pennsylvania, Philadelphia, PA 19104, USA; hongjing.sun@pennmedicine.upenn.edu (H.S.); michele.kim@pennmedicine.upenn.edu (M.M.K.); andreea.dimofte@pennmedicine.upenn.edu (A.D.); keith.cengel@pennmedicine.upenn.edu (K.A.C.); 2 Department of Bioengineering, University of Pennsylvania, Philadelphia, PA 19104, USA; 3 Department of Surgery, University of Pennsylvania, Philadelphia, PA 19104, USA; sunil.singhal@pennmedicine.upenn.edu

**Keywords:** photodynamic therapy, pleural PDT, 3D dosimetry, reactive oxygen species

## Abstract

Photodynamic therapy (PDT) is a promising treatment for pleural malignancies, yet accurate dosimetry remains challenging due to complex cavity geometries and the need to protect surrounding critical structures. The reactive oxygen species ([ROS]_rx_) generated during treatment serve as a direct predictor of therapeutic efficacy. We developed a finite element model using COMSOL Multiphysics to simulate macroscopic photophysical kinetics, using clinical data inputs, including light fluence derived from a navigation system and patient-specific photosensitizer concentrations. Crucially, we integrated a deformable image registration framework to align intra-operative navigation data with pre-treatment CT scans, enabling the calculation of [ROS]_rx_ dose accumulation in critical Organs at Risk (OARs), such as the lung, heart, and esophagus. The model successfully reconstructed 3D [ROS]_rx_ distributions for multiple clinical cases. Point-to-point comparison at 32 detector locations across ten patients showed strong agreement between COMSOL-simulated and clinically calculated [ROS]_rx_ (mean percentage difference 0.6 ± 5.8%), while volume-averaged values differed by −6.0%, reflecting the enhanced spatial coverage of the 3D model relative to discrete sampling. The two-stage deformable registration improved CT-to-navigation surface alignment from HD95 = 4.08 mm to 1.78 mm (56.4% reduction) and MSD = 1.77 mm to 0.68 mm (61.5% reduction), enabling the first patient-specific mapping of [ROS]_rx_ onto OAR structures. This study demonstrates the feasibility of a comprehensive 3D dosimetry system for pleural PDT. By integrating kinetic modeling with deformable registration, we provide a robust platform for evaluating treatment efficacy and ensuring OAR safety, paving the way for eventual integration into treatment planning and real-time feedback.

## 1. Introduction

Photodynamic therapy (PDT) is a light-activated cancer treatment that exploits the selective uptake of a photosensitizer (PS) by tumor tissue to generate cytotoxic reactive oxygen species (ROS) upon illumination in the presence of molecular oxygen [1,2,3,4,5,6]. In the context of malignant pleural mesothelioma (MPM), an aggressive and largely incurable malignancy arising from the pleural lining, PDT has been employed as an adjuvant modality following macroscopic complete resection, with the goal of eradicating microscopic residual disease on the pleural surface [7,8,9]. Early clinical trials established the feasibility of intraoperative pleural PDT and demonstrated encouraging local control rates, cementing its role as a promising component of multimodal treatment strategies for this otherwise refractory disease [10,11,12].

Despite its clinical promise, accurate dosimetry for pleural PDT remains a formidable challenge. The therapeutic outcome of PDT depends on the complex interplay of three fundamental quantities: light fluence rate (ϕ), photosensitizer concentration ([S_0_]), and local oxygen availability ([^3^O_2_]) [13]. Conventional point-based measurements of fluence or drug concentration alone cannot characterize the spatial heterogeneity of the pleural cavity environment, where substantial inter- and intra-patient variability has been documented [14]. The total reacted singlet oxygen concentration ([ROS]_rx_) has emerged as a more mechanistically grounded and clinically predictive dosimetric endpoint than either light fluence or drug dose individually [13,15], with macroscopic kinetic models coupling oxygen, photosensitizer, and singlet oxygen dynamics validated against in vitro and in vivo data. The pleural cavity undergoes substantial geometric remodeling after extrapleural pneumonectomy or pleurectomy, with thoracic structures assuming configurations that differ markedly from preoperative anatomy [16]. Our group recently developed a standardized anatomical coordinate system from intraoperative infrared (IR) navigation data, enabling consistent cross-patient comparison of fluence distributions [17] and simulation of [ROS]_rx_ on anatomically realistic cavity surfaces.

A further challenge in pleural PDT dosimetry concerns the mapping of intraoperatively delivered doses to critical organs at risk (OARs) [18,19]. The thoracic organs adjacent to the pleural cavity, principally the lung, heart, and esophagus, are vulnerable to off-target photodynamic injury, and accurate characterization of dose deposited within these structures is essential for both treatment safety evaluation and long-term outcome analysis [10,20,21]. However, the geometric relationship between preoperative computed tomography (CT) imaging, which provides the anatomical reference for OAR segmentation, and the intraoperative configuration of the pleural cavity is disrupted by the significant soft-tissue deformations that occur during surgery. Rigid registration is insufficient to account for these deformations, necessitating deformable image registration (DIR) approaches capable of capturing the complex nonlinear geometric transformations between the CT and navigation coordinate spaces [22,23].

Deformable image registration is a well-established tool in conventional radiation oncology, where it is employed for adaptive treatment planning, dose accumulation across fractionated courses, and the mapping of functional imaging data onto planning CT volumes [24,25]. Finite element method (FEM)-based DIR approaches, which model tissue deformation using continuum mechanics principles with biomechanically realistic material properties, have demonstrated sub-centimeter accuracy for multi-organ registration tasks in the thoracic and abdominal regions [26,27]. Previous studies have demonstrated the application of FEM-based DIR to the pleural PDT setting and COMSOL Structural Mechanics-based finite element modeling, reporting deformation accuracy consistent with clinical requirements [28]. However, neither study integrated the DIR output with a full three-dimensional ROS dosimetry model capable of computing spatially resolved OAR dose distributions from patient-specific clinical inputs.

The present study addresses this gap by developing and validating a comprehensive, integrated 3D dosimetry framework for pleural PDT that combines macroscopic kinetic ROS modeling with deformable image registration in a unified COMSOL Multiphysics environment. While Penjweini et al. [28] previously demonstrated the feasibility of FEM-based DIR for the pleural cavity, that work did not integrate the registration output with any ROS dosimetry model, leaving the connection between anatomical deformation and therapeutic dose unestablished. Similarly, while our group’s prior clinical ROSED study [14] provided the first comprehensive point-based analysis of [ROS]_rx_ in pleural PDT patients, it was inherently limited to discrete measurement sites and could not provide spatially resolved dose distributions across the pleural surface or map dose onto OAR structures. The present framework is the first to unify these two capabilities: building upon the standardized anatomical coordinate system and macroscopic singlet oxygen kinetic model established in prior work, we implement patient-specific finite element simulations of [ROS]_rx_ distribution using clinical inputs and incorporate a two-stage FEM-based deformable registration pipeline that enables the first patient-level mapping of [ROS]_rx_ dose to critical thoracic organs. This integrated platform provides a robust foundation for comprehensive treatment efficacy analysis and OAR safety evaluation in pleural PDT, with direct implications for future treatment planning and real-time dosimetric feedback systems.

## 2. Methods

### 2.1. Patient Data and Clinical Inputs

**Figure 1:**
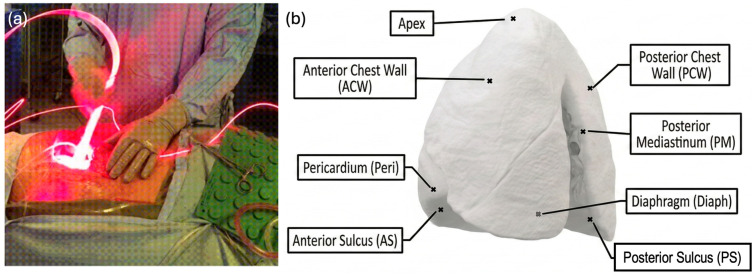
Intraoperative pleural PDT setup and cavity geometry reconstruction: (**a**) Photograph of the intraoperative light delivery procedure, showing the isotropic light source being manually swept across the resected pleural cavity surface. (**b**) Pleural cavity with the eight standardized anatomical detector locations labeled: Apex, Anterior Chest Wall (ACW), Posterior Chest Wall (PCW), Posterior Mediastinum (PM), Pericardium (Peri), Diaphragm (Diaph), Anterior Sulcus (AS), and Posterior Sulcus (PS). These landmarks define a patient-independent coordinate system used for cross-patient registration.

Clinical data from ten patients who underwent pleural PDT at the University of Pennsylvania were retrospectively analyzed under an institutional review board-approved protocol. All patients received Photofrin (porfimer sodium) as the photosensitizer, administered intravenously 24 h prior to surgery at a dose of 2 mg/kg body weight. Intraoperative light delivery was performed using a 630 nm wavelength laser source following macroscopic complete resection of the pleural tumor (Figure 1a) [29]. During treatment, an isotropic light source was swept across the pleural surface, which was measured by 8 isotropic detectors at discrete positions (Figure 1b), with simultaneous tracking of the light source position by an infrared navigation system.

For each patient, three categories of clinical input data were used in the model: pleural cavity geometry reconstructed from the IR navigation point cloud, spatially resolved light fluence rate distributions, and patient-specific photosensitizer concentrations obtained from intraoperative tissue. To enable consistent orientation and cross-patient comparison, all navigation data were registered to a standardized anatomical coordinate system defined by eight anatomical landmarks identified on the reconstructed pleural surface: the Apex, Anterior Chest Wall (ACW), Posterior Chest Wall (PCW), Posterior Mediastinum (PM), Pericardium (Peri), Diaphragm (Diaph), Anterior Sulcus (AS), and Posterior Sulcus (PS) [17]. These landmarks subdivide the pleural cavity into anatomically meaningful regions, facilitating both spatial interpolation of clinical measurements and inter-patient dosimetric comparisons.

### 2.2. Macroscopic Kinetic Model

The generation of [ROS]_rx_ during PDT was simulated using an established macroscopic kinetic model that describes the coupled photophysical dynamics of ground-state oxygen, photosensitizer, and reactive oxygen species. The model is governed by the following system of coupled differential equations:

(1) $$\frac{d{[}^{3}O_{2}]}{dt}+\left(\xi \frac{\phi \left[S_{0}\right]}{{[}^{3}O_{2}]+\beta }\right){[}^{3}O_{2}]=g\left(1-\frac{{[}^{3}O_{2}]}{{{[}^{3}O_{2}]}_{0}}\right)$$

(2) $$\frac{d\left[S_{0}\right]}{dt}+\left(\xi \sigma \frac{\phi \left(\left[S_{0}\right]+\delta \right)}{{[}^{3}O_{2}]+\beta }\right)\left[S_{0}\right]=0$$

(3) $$\frac{d{\left[ROS\right]}_{rx}}{dt}-\left(\xi \frac{\phi \left[S_{0}\right]{[}^{3}O_{2}]}{{[}^{3}O_{2}]+\beta }\right)=0$$

The model parameters carry the following physical meanings, with values adopted from previously published fits to Photofrin-sensitized PDT experimental data [13]: ξ = 3.7 × 10^−3^ (cm^2^ mW^−1^ s^−1^) is the specific oxygen consumption rate, representing the rate of photochemical ^3^O_2_ depletion per unit light fluence rate and per unit [S_0_] under conditions of unlimited oxygen supply and prior to photobleaching; β = 11.9 (µM) is the oxygen quenching threshold, equal to the ratio of monomolecular triplet-state decay to bimolecular triplet quenching by ^3^O_2_, which sets the [^3^O_2_] level below which singlet oxygen production becomes oxygen-limited; δ = 33 (µM) is the low-concentration correction term that accounts for the increased likelihood of singlet oxygen reacting with its parent photosensitizer at vanishing [S_0_]; σ = 7.6 × 10^−5^ (µM^−1^) is the specific photobleaching ratio, defined as the probability ratio of a singlet oxygen molecule reacting with ground-state photosensitizer rather than with a cellular target; and g = 0.76 (µM s^−1^) is the maximum macroscopic oxygen supply rate from the surrounding vasculature in the absence of an oxygen gradient. The initial ground-state oxygen concentration [^3^O_2_]_0_ was set to the standard value of 40 µM. This system of equations was implemented in COMSOL Multiphysics 6.3 with time-dependent solver settings, with the light fluence rate ϕ and photosensitizer concentration [S_0_] entering as spatially distributed input fields interpolated from clinical measurements onto the finite element mesh.

### 2.3. Pleural Cavity Geometry Reconstruction

The intraoperative pleural cavity geometry was reconstructed from position data acquired by an infrared (IR) navigation system during treatment. The treatment wand consisted of a modified endotracheal tube with a balloon applicator tip housing the optical fiber, with nine passive reflective markers attached to allow continuous tracking by an infrared camera (Polaris Spectra, NDI, Waterloo, ON, Canada). As the surgeon swept the wand across the pleural surface during light delivery, the recorded tip positions formed a dense three-dimensional point cloud representative of the cavity boundary at the time of treatment.

The raw point cloud was initially represented in the camera’s intrinsic coordinate system, which varies between treatment sessions depending on camera placement relative to the patient. To enable meaningful cross-patient comparisons, a standardized anatomical coordinate system was applied to all datasets following the procedure. The standardization process involves two key steps: first, a reference orientation is established intraoperatively by holding the treatment wand parallel to the patient’s long axis with the reflective markers directed toward the head, defining a standard coordinate; second, a rotation matrix is applied to align each patient’s cavity reconstruction to this canonical orientation. The eight detector locations were identified within the standardized coordinate system through a combination of automated geometric analysis and manual verification by experienced clinicians and serve as consistent reference points across all patients (Figure 1b) [17].

**Figure 2:**
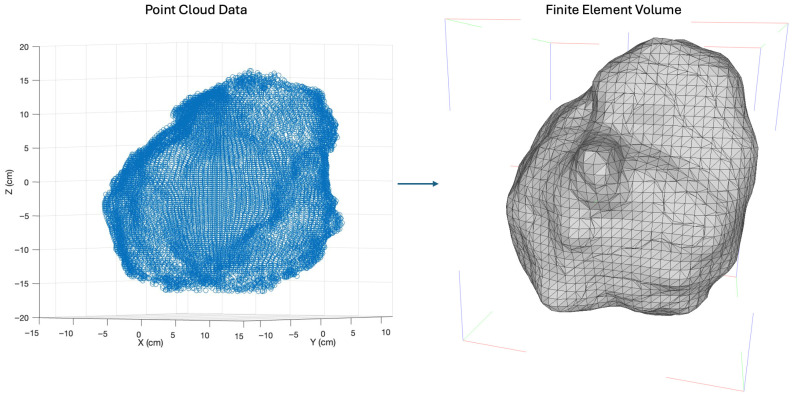
Conversion of intraoperative navigation data to a finite element model: (**Left**) 3D point cloud (blue circles) in the standardized coordinate system. (**Right**) Corresponding COMSOL tetrahedral volume mesh (black lines) generated via direct conversion, preserving patient-specific anatomical features.

The standardized point cloud was subsequently processed using a spherical coordinate transformation algorithm to generate a surface representation of the pleural cavity suitable for finite element modeling. This algorithm efficiently handles the complex and non-convex topology of the pleural space. The resulting triangulated surface was imported into COMSOL Multiphysics 6.3 using a direct conversion pipeline that preserves critical anatomical concavities. Conventional surface interpolation methods, such as spline fitting or coarse-grid tessellation, were found to introduce systematic smoothing errors that obliterate clinically relevant geometric features; the direct import approach avoids this by constructing the COMSOL-compatible tetrahedral volume mesh directly from the navigation-derived triangulation without intermediate interpolation (Figure 2) [30,31]. The final finite element volume mesh served as the computational domain on which the macroscopic kinetic model was solved.

### 2.4. Light Fluence and Photosensitizer Distribution

The local light fluence ϕ (J/cm^2^) at each surface point was calculated using a semi-empirical model accounting for both direct illumination and multiply scattered photons:

(4) $$\phi (r,t)=\left(\frac{S}{4\pi r(t{)}^{2}}+b\right)\cdot CF(t)$$

where S is the instantaneous source power (mW), r(t) is the distance from the moving isotropic source to each surface point at time t, b is an empirically determined scattered light constant, and CF(t) is a dual correction factor accounting for tissue optical properties and source-detector geometry.

The in vivo Photofrin concentration [S_0_] was measured simultaneously at each of the isotropic detector positions (Medlight SA, Ecublens, Switzerland) sutured to the standardized anatomical landmarks. Photofrin fluorescence spectra excited at 630 nm were acquired throughout treatment and decomposed via singular value decomposition (SVD) to isolate the Photofrin contribution from autofluorescence. To obtain absolute photosensitizer concentrations, the raw fluorescence amplitude was corrected for tissue optical property distortion using an empirical correction function. This approach has been shown to recover absolute Photofrin concentration accurately from in vivo pleural measurements despite large inter- and intra-patient variations in tissue optical properties [29].

**Figure 3:**
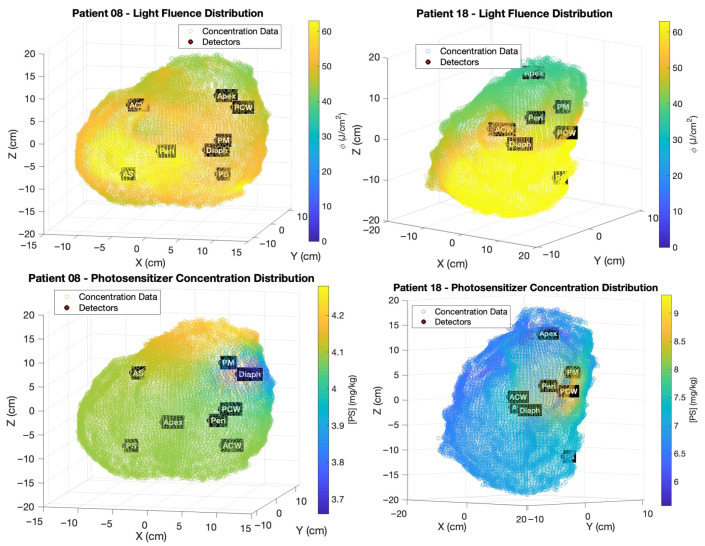
Calculated light fluence distribution ϕ (J/cm^2^) (**top row**) and spatially interpolated Photofrin concentration [S_0_] (mg/kg) (**bottom row**) mapped onto the pleural cavity surface point cloud for two representative patients (Patient 08, (**left**); Patient 18, (**right**)). Marked inter-patient heterogeneity is observed in both distributions, motivating patient-specific 3D dosimetry.

The resulting discrete measurements of [S_0_] were spatially interpolated onto all surface nodes of the patient’s COMSOL tetrahedral mesh using inverse-distance weighting (IDW), assigning weights inversely proportional to the Euclidean distance from each mesh node to each detector site [30]. This produced spatially heterogeneous, patient-specific distributions of [S_0_] as continuous input fields for the kinetic model. Representative distributions of ϕ and [S_0_] for two patients (Patient 08 and Patient 18) are shown in Figure 3, illustrating the substantial inter-patient variability in both light delivery and photosensitizer uptake across the pleural surface.

### 2.5. Pre-Treatment Volume Segmentation and Import

**Figure 4:**
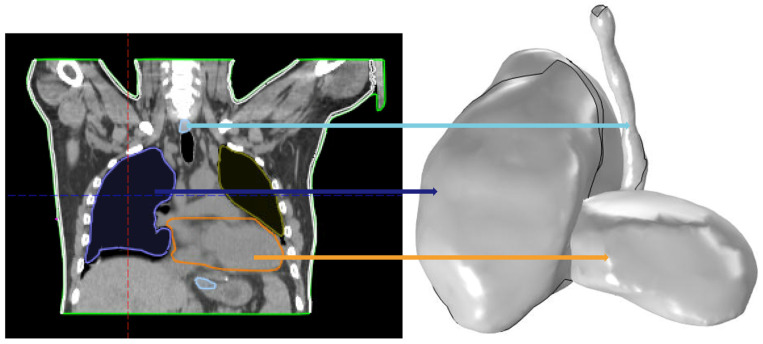
Pre-treatment OAR segmentation and 3D geometry import: (**Left**) Coronal CT slice with manually contoured structures: heart (orange), esophagus (light blue), and lung (navy). (**Right**) Corresponding 3D solid geometries reconstructed in COMSOL Multiphysics from exported DICOM RT structure sets, serving as finite element domains for biomechanical deformation modeling.

Planning CT images acquired prior to surgery were used to delineate thoracic OARs using the Eclipse treatment planning system (Varian Medical Systems, Palo Alto, CA, USA) following established contouring guidelines for thoracic structures [32,33]. The resulting DICOM RT structure sets were parsed in MATLAB 2022b using custom routines that load the CT image volume and extract per-slice contour point sets for each structure of interest. At each axial level, multiple contours were evaluated, and artifact contours were rejected based on two criteria: minimum fractional area relative to the largest contour at that slice (threshold: 5%) and minimum centroid separation from the primary contour (threshold: 2 cm), with the signed enclosed area computed by the shoelace formula. The cleaned contour stacks were assembled into closed 3D surface meshes and exported as STL files, then imported into COMSOL Multiphysics as solid geometries and discretized into tetrahedral finite element domains (Figure 4). This pipeline generalizes to any OAR delineated in the treatment planning system, enabling patient-specific biomechanical modeling of whichever structures are relevant to a given surgical case.

### 2.6. Deformable Image Registration Framework

**Figure 5:**
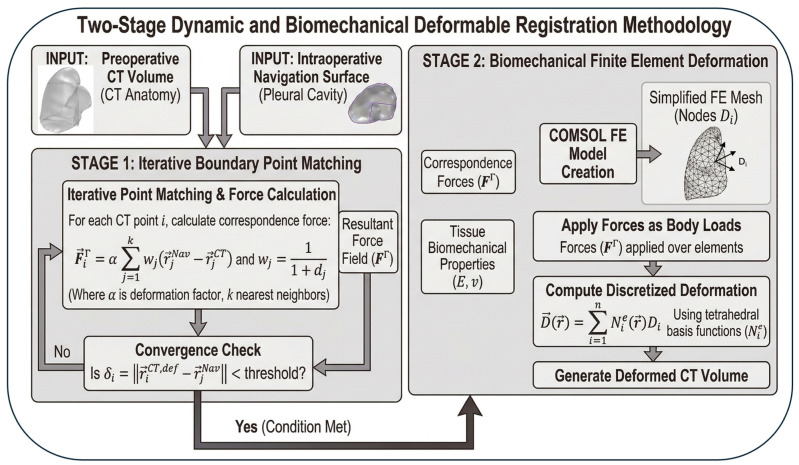
Two-stage dynamic and biomechanical deformable image registration methodology. Allows indicate the flow of input parameters or flow direction choices (with “Yes” or “No” of convergence check).

A two-stage deformable image registration (DIR) framework was implemented in COMSOL Multiphysics to spatially map the preoperative CT anatomy onto the intraoperative pleural cavity surface reconstructed during PDT delivery. A schematic overview of the two-stage pipeline is shown in Figure 5: Stage 1 establishes a geometric correspondence between the preoperative CT anatomy and the intraoperative navigation surface through iterative boundary point matching driven by inverse-distance-weighted force vectors. Stage 2 propagates this surface correspondence into the OAR volume by treating the CT structures as linear elastic continua and solving for the displacement field that minimizes total mechanical energy under the Stage 1 force loads. The output is a fully deformed CT geometry registered to the intraoperative navigation surface, on which the macroscopic kinetic model from Section 2.2 is solved to obtain spatially resolved [ROS]_rx_ on both the treated pleural surface and the registered OAR structures.

The intraoperative navigation surface serves as the fixed reference, while the CT-derived OAR geometries from Section 2.5 constitute the deformable moving volumes. The registration minimizes the total mechanical energy over the CT volume domain Ω [26,34]:

(5) $$\Delta =\int_{\Omega }\sigma^{T}\varepsilon \mathrm{dV}+\int_{\Omega }\overset{⃑}{F}\cdot \overset{⃑}{D}\mathrm{dV}$$

where σ is the Cauchy stress tensor, ε is the strain tensor, F is the external force field, and D is the displacement field.

In Stage 1, iterative boundary point matching establishes a spatial correspondence between the CT anatomy and the navigation surface. For each CT boundary point i, a weighted correspondence force vector is computed from the k nearest navigation surface neighbors:

(6) $$\overset{⃑}{F_{i}}\;=\;\alpha \sum_{j=1}^{k}w_{j}({\overset{⃑}{r}}_{j}^{Nav}-{\overset{⃑}{r}}_{i}^{CT}),{\;w}_{j}=\frac{1}{1+d_{j}}$$

where α is a deformation scaling factor, and d_j is the Euclidean distance to the j-th neighbor. The resultant force field F^Γ is updated iteratively until the boundary residual satisfies the convergence criterion:

(7) $$\delta_{i\;}=\;∥{\overset{⃑}{r}}_{i}^{CT,def}-{\overset{⃑}{r}}_{j}^{Nav}∥\;<\;\mathrm{threshold}$$

where ε is a sub-centimeter displacement threshold.

In Stage 2, the OAR geometries are discretized into tetrahedral finite element domains within COMSOL’s Structural Mechanics module and assigned linear elastic material properties characterized by Young’s modulus E and Poisson’s ratio ν [35]. The correspondence force field $\overset{⃑}{F_{i}}$ from Stage 1 is applied as distributed body loads, and the discretized deformation field is computed as

(8) $$\overset{⃑}{D}(\overset{⃑}{r})=\sum_{i=1}^{n}N_{i}^{e}(\overset{⃑}{r})\overset{⃑}{D_{i}}$$

where $N_{i}^{e}(\overset{⃑}{r})$ are the tetrahedral basis functions, and n is the number of nodes per element. The deformed CT volume with registered OAR positions is then used to evaluate [ROS]_rx_ at the treated volume as well as in other essential organs.

Registration accuracy was quantified using two complementary surface-based metrics computed between the deformed CT boundary point cloud and the navigation reference surface. The 95th-percentile Hausdorff distance (HD95) reports the 95th percentile of the symmetric minimum-distance distribution between the two-point sets, providing a robust measure of worst-case boundary mismatch that excludes single-point outliers. The mean surface distance (MSD) reports the mean of the same symmetric distance distribution and characterizes typical boundary agreement. Both metrics were computed before and after registration to quantify the geometric improvement achieved by the two-stage DIR pipeline.

### 2.7. Model Validation

The integrated 3D ROS dosimetry framework was validated through two complementary approaches: a summary-level volume-averaged comparison and a point-to-point spatial comparison between COMSOL-simulated and clinically calculated [ROS]_rx_ at discrete detector locations.

For the volume-averaged comparison, the mean [ROS]_rx_ simulated by the COMSOL macroscopic kinetic model across the pleural surface was compared against the mean clinical [ROS]_rx_ values computed from the same measured light fluence inputs and photochemical parameters using the same coupled kinetic equations (Equations (1)–(3)), with tissue oxygenation estimated from the macroscopic model assuming an initial oxygen concentration of 40 µM [14].

For the point-to-point comparison, the 3D [ROS]_rx_ distribution computed by COMSOL 6.3 was sampled at the spatial coordinates corresponding to the eight isotropic detector locations within the reconstructed pleural cavity geometry (Figure 1b). These detector-coincident COMSOL values were directly compared against the clinically calculated [ROS]_rx_ at those same eight anatomical sites for each patient. Agreement was quantified by the mean percentage difference and standard deviation across all sites and patients.

It should be noted that the clinical [ROS]_rx_ values used in both comparisons are themselves derived from the same macroscopic kinetic model (Equations (1)–(3)), using the same measured inputs of light fluence and photosensitizer concentration. The validation therefore demonstrates the numerical accuracy and spatial fidelity of the COMSOL finite element implementation relative to the established point-based clinical calculation, rather than providing an independent experimental benchmark against a biological or physical ground truth. True independent validation would require biological surrogates of singlet oxygen dose, such as tissue damage scores, photobleaching depth profiles, or ex vivo cell viability measurements correlated with spatially resolved [ROS]_rx_, and remains an important direction for future work.

## 3. Results

### 3.1. Deformable Image Registration

**Figure 6:**
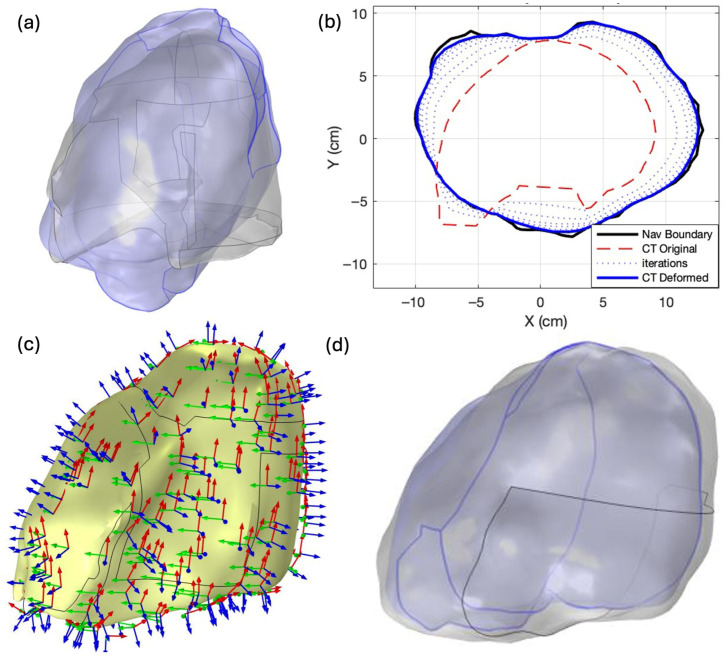
Deformable image registration process and results for a representative patient: (**a**) Initial 3D overlay of the intraoperative navigation volume (blue) and CT-derived lung volume (gray) prior to registration. (**b**) Cross-sectional view in the X–Y plane illustrating iterative convergence: navigation boundary (black solid), original CT contour (red dashed), intermediate iterations (blue dotted), and final deformed CT contour (blue solid). (**c**) Von Mises stress distribution (N/m^2^) with three-dimensional displacement vectors (red: x, green: y, blue: z) computed during Stage 2 biomechanical deformation. (**d**) Final deformed CT volume (gray) overlaid with the navigation volume (blue), demonstrating successful geometric alignment.

The two-stage deformable image registration framework successfully aligned the preoperative CT-derived lung geometry to the intraoperative navigation surface. Figure 6 illustrates the registration process and outcome for a representative case. The initial overlay of the raw navigation volume (blue) and the CT-derived lung volume (gray) revealed substantial geometric mismatch, reflecting the significant soft-tissue deformation between preoperative imaging and the intraoperative pleural cavity configuration (Figure 6a). Figure 6b presents a cross-sectional view in the X–Y plane demonstrating the iterative boundary point matching process (Stage 1): the navigation boundary (black solid) serves as the fixed target, the original CT contour (red dashed) represents the initial moving boundary, intermediate iterations (blue dotted) show progressive refinement, and the final deformed CT contour (blue solid) demonstrates close agreement with the navigation target. Figure 6c depicts the von Mises stress distribution (N/m^2^) computed during Stage 2 biomechanical deformation in COMSOL, with the displacement vector field overlaid on the CT volume. Figure 6d presents the final deformed CT volume (gray) overlaid with the navigation volume (blue), confirming successful geometric correspondence following the two-stage registration. This registered geometry establishes the spatial foundation for evaluating [ROS]_rx_ dose deposition on OAR structures.

Quantitative evaluation confirms the visual agreement shown in Figure 6. Prior to registration, the CT-derived lung surface differed from the intraoperative navigation reference by HD95 = 4.08 mm and MSD = 1.77 mm, reflecting the substantial geometric distortion introduced by surgical resection. Following the two-stage deformable registration, both metrics improved substantially to HD95 = 1.78 mm and MSD = 0.68 mm, corresponding to reductions of 56.4% and 61.5%, respectively. These post-registration values are consistent with the sub-centimeter accuracy reported for FEM-based DIR in thoracic and abdominal applications [26,27], confirming that the framework achieves boundary alignment sufficient for downstream OAR dose mapping.

### 3.2. Three-Dimensional [ROS]_rx_ Distribution

**Figure 7:**
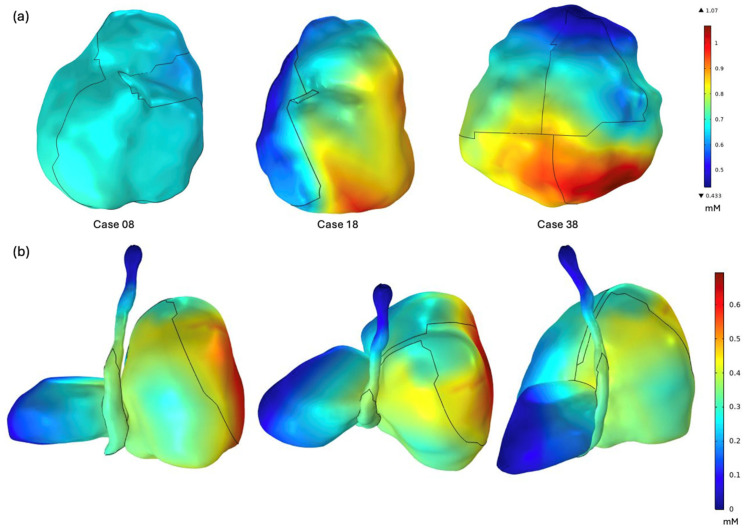
Three-dimensional [ROS]_rx_ dose distributions: (**a**) Cumulative [ROS]_rx_ (mM) mapped onto the treated lung surface for three representative cases (Case 08, Case 18, and Case 38), each with a patient-specific color scale. (**b**) [ROS]_rx_ distribution for a single case shown from lateral (**left**), superior (**center**), and posterior (**right**) views, mapped onto the treated lung and registered OAR structures, including the esophagus and heart.

The COMSOL macroscopic kinetic model was solved for all patients, yielding spatially resolved three-dimensional [ROS]_rx_ distributions across the pleural cavity surface. Figure 7a presents the cumulative [ROS]_rx_ dose mapped onto the treated lung surface for three representative cases (Case 08, Case 18, and Case 38). Marked spatial heterogeneity is evident across all three cases, with high-dose regions (red) and low-dose regions (blue) distributed non-uniformly across the pleural surface. The inter-patient variability in both the absolute dose range and the spatial dose pattern reflects differences in cavity geometry, light delivery trajectories, and photosensitizer uptake, underscoring the necessity of patient-specific 3D dosimetry over uniform-dose assumptions.

Figure 7b demonstrates the [ROS]_rx_ distribution for a single representative case viewed from three orientations (lateral, superior, and posterior), with the dose mapped onto both the treated lung and the registered OAR structures following deformable image registration. Substantial dose gradients are observed across the OAR surfaces, with regions adjacent to high-fluence areas of the pleural cavity receiving elevated [ROS]_rx_ while more distant portions remain at near-zero levels. This spatially resolved OAR dose mapping, enabled by the deformable registration framework described in Section 3.1, provides information previously unattainable with discrete point dosimetry and is critical for identifying potential regions of off-target photodynamic toxicity.

### 3.3. Volume-Averaged Dosimetric Comparison

Table 1 summarizes the comparison between COMSOL-simulated volume-averaged [ROS]_rx_ and clinically calculated mean [ROS]_rx_ values for each of the ten patients. The COMSOL values represent the surface-averaged cumulative [ROS]_rx_ computed by the finite element kinetic solver across the entire pleural cavity mesh, while the clinical values were derived from the mean of [ROS]_rx_ calculated at discrete isotropic detector locations using the same coupled kinetic equations (Equations (1)–(3)). Across the cohort, the COMSOL-simulated mean [ROS]_rx_ was 0.68 ± 0.11 mM compared to the clinical mean of 0.73 ± 0.13 mM, yielding an overall mean percentage difference of −6.0%. Individual case differences ranged from −28.8% to 18.3%, with both positive and negative deviations observed.

### 3.4. Spatial Validation at Detector Locations

To evaluate the spatial fidelity of the 3D dosimetry model beyond volume-averaged metrics, a point-to-point comparison was performed at discrete isotropic detector locations for each patient. Table 2 presents the COMSOL-simulated [ROS]_rx_ alongside the clinically calculated [ROS]_rx_ at available detector sites, together with the percentage difference at each measurement point and per-case mean ± standard deviation. The number of available detector sites varied across patients (two to four per case) depending on the clinical measurement configuration, yielding a total of 32 comparison points across the ten-patient cohort. The site-by-site comparison demonstrates strong agreement between the COMSOL model and clinical values, with an overall mean percentage difference of 0.6 ± 5.8% and individual point deviations ranging from −8.5% to 13.2%. Per-case mean differences were consistently small, ranging from −6.2% (Case 12) to 5.8% (Case 37), with no systematic directional bias. Cases with near-perfect point-to-point agreement confirm that the finite element model faithfully reproduces the clinical dosimetric calculation when the spatial inputs are well characterized. Overall, the close spatial agreement validates the 3D COMSOL model as a reliable tool for reconstructing [ROS]_rx_ distributions across the pleural surface.

## 4. Discussion

This study presents the first integrated 3D dosimetry framework for pleural PDT that combines macroscopic kinetic [ROS]_rx_ modeling with FEM-based deformable image registration within a unified COMSOL Multiphysics environment. The framework addresses a critical gap that neither of the two most closely related prior studies could resolve individually. Penjweini et al. [28] demonstrated that FEM-based DIR could successfully align CT-derived pleural anatomy to intraoperative geometry but did not couple the registration output to any dosimetric model, leaving the spatial distribution of therapeutic dose on OAR structures uncharacterized. Conversely, our group’s comprehensive clinical ROSED study [14] established the feasibility and clinical relevance of [ROS]_rx_ as a dosimetric endpoint in pleural PDT patients but was confined to point measurements at eight discrete detector locations and could not resolve the spatial heterogeneity of dose across the full pleural surface or quantify OAR exposure. The present framework closes both gaps simultaneously: by solving the macroscopic kinetic model on the deformed finite element geometry, spatially resolved [ROS]_rx_ distributions are obtained across the entire pleural surface and projected onto registered OAR structures, enabling dosimetric information that is qualitatively beyond what either prior approach could provide.

The deformable image registration results in Section 3.1 demonstrate that the two-stage approach can successfully align preoperative CT-derived lung geometry to the intraoperative navigation surface despite the substantial geometric distortion introduced by surgical resection. Previous work demonstrated the feasibility of FEM-based DIR for the pleural cavity using COMSOL but did not integrate the registration output with a spatially resolved ROS dosimetry model [28]. Our framework extends this prior work by establishing the deformed geometry as the computational domain on which the macroscopic kinetic model is solved, enabling direct evaluation of [ROS]_rx_ on registered OAR surfaces. The extensibility of this framework to other patients and additional OAR structures delineated in the treatment planning system has been demonstrated.

The volume-averaged comparison in Section 3.3 showed an overall mean percentage difference of −6.0% between the COMSOL-simulated and clinically calculated [ROS]_rx_ values. This discrepancy is expected and attributable to a fundamental difference in spatial sampling: the COMSOL model computes [ROS]_rx_ continuously over the entire pleural surface mesh, including regions between and beyond the eight discrete detector locations, whereas the clinical calculation averages only the values at those sparse measurement sites. In cases with relatively uniform dose distributions (e.g., Case 16: −3.8%), the discrete clinical measurements adequately approximate the volume average, while cases with greater spatial heterogeneity (e.g., Case 14: −28.8%) show larger discrepancies as the eight-point sampling increasingly underrepresents the true dose distribution.

In contrast, the point-to-point comparison in Section 3.4 demonstrated strong spatial agreement, with an overall mean percentage difference of only 0.6 ± 5.8% across 32 measurement points. This result confirms that at locations where the COMSOL model and clinical calculation share identical spatial inputs, the finite element solver faithfully reproduces the established clinical dosimetric calculation. The small residual differences at individual sites, ranging from −8.5% to 13.2%, likely reflect the sensitivity of the inverse-distance-weighted interpolation to local geometric complexity, particularly in anatomically recessed areas such as the sulci, where sparse detector coverage limits interpolation accuracy. It is important to recognize that this agreement, while confirming the numerical fidelity of the finite element solver, reflects the internal self-consistency of the computational framework rather than independent experimental validation. Since both the COMSOL-simulated and clinical [ROS]_rx_ values are derived from the same kinetic equations and the same measured inputs, the close agreement is an expected consequence of solver accuracy rather than evidence of biological ground-truth validity. The volume-averaged divergence observed in Table 1, by contrast, arises from a genuine difference in spatial sampling strategy between the 3D model and sparse point measurements and is therefore a more informative indicator of the added value of the 3D framework over conventional dosimetry.

The COMSOL-simulated [ROS]_rx_ distributions revealed substantial spatial heterogeneity across the ten-patient cohort, underscoring the limitations of uniform-dose assumptions in pleural PDT. As summarized in Table 1, the volume-averaged [ROS]_rx_ ranged from 0.43 mM (Case 12) to 0.81 mM (Case 38), representing up to 1.9-fold variations between patients, while intra-patient site-to-site differences reached up to 2.4-fold within individual pleural cavities (e.g., Case 38: 0.43 mM at the Apex to 1.04 mM at the PS, Table 2). These heterogeneities arise from the combined influence of patient-specific cavity geometry, non-uniform light delivery trajectories, and spatially variable Photofrin uptake, all of which interact through the nonlinear coupled kinetic equations (Equations (1)–(3)) to produce dose distributions that cannot be predicted from any single parameter alone. The magnitude of this spatial variation has direct clinical implications: regions receiving [ROS]_rx_ below the reported singlet oxygen threshold dose of approximately 0.56 mM for Photofrin-mediated PDT may harbor undertreated microscopic residual disease, while focal high-dose regions on OAR surfaces identified through the deformable registration framework may signal a risk of off-target photodynamic injury. Conventional point dosimetry, which samples only eight discrete locations within the cavity, is inherently unable to capture these gradients, as evidenced by the divergence between the volume-averaged COMSOL values and the sparse-sampling clinical mean. The 3D dosimetry framework thus provides a more complete characterization of the therapeutic dose landscape, enabling identification of both undertreated and overexposed regions that would otherwise remain invisible to discrete monitoring.

Several limitations of the current framework should be acknowledged. The photosensitizer concentration is interpolated from a maximum of eight discrete detector measurements using IDW, which may not fully capture steep concentration gradients in regions far from measurement sites. Higher-density intraoperative sampling or model-based interpolation schemes could improve spatial resolution. A related concern applies to tissue oxygenation: the macroscopic kinetic model assumes a uniform initial oxygen concentration of 40 µM across the pleural surface, whereas in reality oxygenation likely varies spatially, particularly in regions with compromised vascularity following surgical resection. On the biomechanical side, Stage 2 of the DIR employs a linear elastic constitutive model with isotropic Young’s modulus and Poisson’s ratio for all tissues. This formulation simplifies the true mechanical behavior of thoracic soft tissues in three respects: it neglects the nonlinear stress–strain response that emerges at larger strains, the anisotropy introduced by fiber orientation in lung parenchyma and pericardium, and the viscoelastic time-dependence governing tissue relaxation under sustained loading. Consequently, the model may underestimate local strain concentrations near stiff–soft tissue interfaces and cannot capture creep or stress-relaxation effects over the treatment time scale. The formulation was nonetheless retained on two grounds: prior FEM-based DIR studies in thoracic and abdominal applications have employed equivalent linear elastic models and reported sub-centimeter accuracy comparable to that achieved here [36,37], and the magnitude of surgical deformation in the present cohort (pre-registration HD95 = 4.08 mm) lies within the small-strain regime where linear elastic theory is most reliable. Hyperelastic or viscoelastic constitutive models may be warranted in future extensions involving larger deformations, longer integration times, or organ-specific mechanical fidelity. More broadly, the current framework operates retrospectively, and integration into a real-time intraoperative dosimetry system will require substantial reductions in computational time, potentially through model order reduction or GPU-accelerated solvers. The present validation cohort of ten patients, while sufficient to demonstrate technical feasibility and internal consistency of the integrated framework, is modest in size and limits the statistical power of any direct correlation between dosimetric quantities and clinical outcomes. Expanding the analysis to a larger patient cohort and prospectively tracking long-term outcomes represents the most important future validation strategy. Such correlation would provide the independent biological benchmark currently lacking from the present internal consistency validation and would establish whether the spatially resolved [ROS]_rx_ distributions reported here are predictive of clinical efficacy and toxicity at the patient level.

A further limitation concerns the treatment of tissue oxygenation within the macroscopic kinetic model. The initial ground-state oxygen concentration [^3^O_2_]_0_ was set to a uniform value of 40 µM across the pleural surface, consistent with the standard assumption employed in prior macroscopic singlet oxygen modeling for Photofrin-mediated PDT [13,14]. However, clinical data from our group’s comprehensive ROSED study [14] demonstrate that intraoperative pleural oxygenation is highly dynamic and spatially heterogeneous: measured [^3^O_2_] exhibited acute fluctuations directly correlated with local fluence rate, with maximum inter-patient and intra-patient variations of 3.8-fold and 3.0-fold, respectively, across measurement sites. A direct consequence is that the macroscopic model is likely to overestimate [ROS]_rx_ in regions of transient hypoxia and underestimate the degree of spatial heterogeneity in the final dose distribution. Indeed, when the same macroscopic modeling approach was applied to estimate [^3^O_2_] from fluence rate data alone, the resulting [ROS]_rx_,_calc2_ overestimated directly measured [ROS]_rx_,_meas_ by 44 ± 66% on average [14]. While our group has developed a clinical method for intraoperative [^3^O_2_] monitoring via diffuse correlation spectroscopy (DCS) of tumor blood flow [14], the number of DCS measurement sites achievable within the operative field is limited by surgical logistics, precluding its routine integration into the present framework. Incorporating patient-specific, time-resolved oxygenation measurements into the COMSOL kinetic solver, as the DCS infrastructure matures and expands toward more measurement channels, represents a high-priority direction for future work that would substantially improve the quantitative accuracy of the 3D [ROS]_rx_ distributions reported here.

Beyond oxygenation, a brief sensitivity analysis of the remaining model assumptions provides further context for the uncertainty in the reported [ROS]_rx_ distributions. Regarding the optical correction factor CF applied to recover absolute Photofrin concentration from fluorescence spectra, clinical measurements across 78 sites in 20 patients demonstrated CF values ranging from 0.54 to 2.57, a 4.7-fold variation driven by tissue absorption coefficients ranging from 0.08 to 0.72 cm^−1^ and reduced scattering coefficients from 4.97 to 16.6 cm^−1^ [29]. This correction was applied on a per-site basis in the present study using measured optical properties, meaning the uncertainty in [S_0_] at each detector location is governed by the accuracy of the diffuse reflectance fitting rather than the full range of CF variability. Regarding light fluence, GPU-accelerated Monte Carlo simulations across the same optical property range observed in pleural tissues demonstrated that scattered fluence rate per source power varies by approximately 2–3-fold across this range and that anatomically realistic pleural cavities exhibit a spatial coefficient of variation of ~50% compared with ~5% in idealized spherical geometries [38]. The semi-empirical fluence model used here incorporates both direct and scattered components with an empirically determined scatter constant b, which has been validated to within 0.9–12.8% of measured values. Finally, the IDW interpolation used to distribute the eight discrete [S_0_] measurements across the full pleural mesh may underrepresent steep local gradients, particularly in anatomically recessed regions such as the sulci, contributing to the residual point-to-point discrepancies observed in Table 2 (up to 13.2%). Collectively, these analyses indicate that oxygenation heterogeneity remains the dominant source of quantitative uncertainty, while the optical correction and light fluence components are substantially mitigated by site-specific measurements and validated models, respectively.

Despite these limitations, the integrated framework provides a robust foundation for comprehensive treatment evaluation in pleural PDT. The ability to generate patient-specific, spatially resolved [ROS]_rx_ distributions across both the target pleural surface and adjacent OARs represents a significant step toward the development of dose-volume histogram-based treatment assessment analogous to that employed in conventional radiation therapy. Future work will focus on extending the multi-organ deformable registration to the full patient cohort, incorporating dose-volume constraints for OAR safety evaluation, and exploring the integration of this framework with real-time navigation feedback to enable prospective treatment guidance.

Beyond demonstrating technical feasibility, the integrated 3D dosimetry framework carries direct implications for clinical decision-making in pleural PDT. The ability to reconstruct spatially resolved [ROS]_rx_ distributions across the entire pleural surface, rather than sampling at eight discrete points, enables the identification of focal undertreated regions where [ROS]_rx_ falls below the Photofrin singlet oxygen threshold dose. Such regions, which would be invisible to conventional point dosimetry, may correspond to sites of microscopic residual disease at elevated risk for local recurrence. Conversely, the mapping of [ROS]_rx_ onto registered OAR surfaces provides a quantitative basis for post-treatment safety evaluation: localized high-dose regions on the heart, esophagus, or lung surface can be identified and correlated with clinical toxicity outcomes in future prospective studies, establishing dose–response relationships analogous to those that underpin OAR constraints in conventional radiotherapy. While definitive OAR-specific [ROS]_rx_ toxicity thresholds for pleural PDT have not yet been established, prior clinical experience provides initial anchors for hypothesis generation. Documented OAR toxicities in pleural PDT and adjacent cavitary applications include esophageal perforation and stricture, atrial fibrillation, and post-treatment pneumonitis, which have historically been reported in terms of delivered light fluence rather than [ROS]_rx_ [39,40]. By enabling spatially resolved [ROS]_rx_ mapping onto registered OAR surfaces, the present framework provides the dosimetric substrate needed to retrospectively correlate localized OAR exposures with clinical toxicity outcomes and to derive structure-specific tolerance values empirically from prospective outcome data. Taken together, these capabilities position the framework as a tool for retrospective treatment adequacy assessment and OAR safety profiling, two functions currently absent from clinical pleural PDT practice. The pathway toward real-time intraoperative implementation builds incrementally on this foundation. In the near term, pre-computed patient-specific finite element meshes and pre-loaded photophysical parameters can reduce intraoperative computation to the kinetic solver alone, which operates on timescales compatible with treatment delivery. Accelerated solvers, including GPU-based or reduced-order model implementations, could further compress computation times toward the sub-minute range required for practical intraoperative feedback. Ultimately, integration with the existing infrared navigation infrastructure, which already provides real-time source position tracking and fluence accumulation, would enable continuous [ROS]_rx_ mapping updated as treatment progresses, allowing the surgeon to identify and remediate undertreated regions before the chest is closed. While these steps require further engineering development and prospective clinical validation, the present framework establishes the computational and registration foundation upon which such a real-time system can be built.

## 5. Conclusions

This study demonstrates the feasibility of a comprehensive 3D dosimetry framework for pleural PDT that integrates macroscopic kinetic [ROS]_rx_ modeling with FEM-based deformable image registration in a unified COMSOL Multiphysics environment. The framework successfully reconstructed spatially resolved [ROS]_rx_ distributions across the pleural cavity surface for a ten-patient cohort using patient-specific clinical inputs, revealing marked spatial heterogeneity that underscores the limitations of conventional point-based dosimetric approaches. Point-to-point validation at discrete detector locations confirmed a strong agreement between the COMSOL model and established clinical calculations, while the observed divergence in volume-averaged comparisons reflects the enhanced spatial coverage of the 3D model relative to sparse discrete sampling. The two-stage deformable registration pipeline successfully aligned CT-derived anatomy to the intraoperative navigation geometry, enabling the first mapping of [ROS]_rx_ dose onto OAR structures in pleural PDT. This integrated platform provides a foundation for patient-specific treatment evaluation, OAR safety assessment, and future development toward real-time intraoperative dosimetric guidance.

## Figures and Tables

**Table 1:** Comparison of COMSOL-simulated volume-averaged [ROS]_rx_ and clinically calculated mean [ROS]_rx_ at discrete detector locations for the ten-patient cohort. Percentage difference quantifies the deviation between the two methods.

Case No.	COMSOL-Simulated [ROS]_rx_ * (mM)	Clinical [ROS]_rx_ ^#^ (mM)	Percentage Difference
08	0.67 ± 0.03	0.64 ± 0.10	4.69%
12	0.43 ± 0.08	0.58 ± 0.23	−26.6%
14	0.71 ± 0.04	0.99 ± 0.17	−28.8%
16	0.77 ± 0.04	0.80 ± 0.18	−3.8%
17	0.67 ± 0.07	0.75 ± 0.10	−10.8%
18	0.72 ± 0.11	0.67 ± 0.14	7.3%
20	0.68 ± 0.08	0.90 ± 0.11	−24.0%
27	0.59 ± 0.06	0.67 ± 0.07	−11.6%
37	0.71 ± 0.09	0.60 ± 0.18	18.3%
38	0.81 ± 0.12	0.70 ± 0.26	15.7%
Mean ± SD	0.68 ± 0.11	0.73 ± 0.13	−6.0%

* Volume-averaged value. ^#^ Mean value from clinically calculated [ROS]_rx_ at discrete locations.

**Table 2:** Point-to-point comparison of COMSOL-simulated and clinically calculated [ROS]_rx_ (mM) at discrete isotropic detector locations for all ten patients. Per-case mean ± standard deviation and percentage difference are reported. Overall mean percentage difference across 32 measurement points: 0.6 ± 5.8%. The **bold** mean ± SD values in Table 2 is different from the mean ± SD values in Table 1 as it is only the sum of the points in the table (Table 2) rather than for all points (Table 1).

Case No.	Site	COMSOL-Simulated [ROS]_rx_	Clinical [ROS]_rx_	Percentage Difference
08	Apex	0.65	0.71	−8.5%
	PCW	0.60	0.57	5.3%
	Mean ± SD	**0.62 ± 0.04**	**0.64 ± 0.10**	
12	Apex	0.39	0.42	−7.1%
	PM	0.71	0.75	−5.3%
	Mean ± SD	**0.55 ± 0.23**	**0.58 ± 0.23**	
14	PS	1.15	1.11	3.6%
	Peri	0.82	0.87	−5.7%
	Mean ± SD	**0.98 ± 0.23**	**0.99 ± 0.17**	
16	Apex	0.88	0.93	−5.4%
	PCW	0.70	0.67	4.5%
	Mean ± SD	**0.79 ± 0.13**	**0.80 ± 0.18**	
17	Apex	0.71	0.72	−1.4%
	PCW	0.80	0.85	−5.9%
	PM	0.58	0.62	−6.5%
	ACW	0.85	0.80	6.2%
	Mean ± SD	**0.73 ± 0.12**	**0.75 ± 0.10**	
18	PS	0.72	0.73	−1.4%
	Apex	0.63	0.64	−1.6%
	PCW	0.89	0.82	8.5%
	ACW	0.49	0.48	2.1%
	Mean ± SD	**0.68 ± 0.17**	**0.67 ± 0.14**	
20	PS	1.00	0.94	6.4%
	Apex	0.75	0.75	0.0%
	PCW	0.99	1.02	2.9%
	PM	0.91	0.88	3.4%
	Mean ± SD	**0.91 ± 0.12**	**0.90 ± 0.11**	
27	Apex	0.71	0.67	6.0%
	PM	0.59	0.58	1.7%
	Peri	0.68	0.72	−5.6%
	ACW	0.70	0.72	−2.8%
	Mean ± SD	**0.67 ± 0.05**	**0.67 ± 0.07**	
37	Apex	0.66	0.60	10.0%
	PCW	0.89	0.82	8.5%
	PM	0.55	0.60	−8.3%
	ACW	0.43	0.38	13.2%
	Mean ± SD	**0.63 ± 0.20**	**0.60 ± 0.18**	
38	PS	1.04	1.04	0.0%
	Apex	0.43	0.44	2.3%
	PM	0.58	0.59	1.7%
	ACW	0.80	0.75	6.7%
	Mean ± SD	**0.72 ± 0.26**	**0.70 ± 0.26**	

## Data Availability

The original contributions presented in this study are included in the article. Further inquiries can be directed to the corresponding author.

## Abbreviations

The following abbreviations are used in this manuscript:

PDT	Photodynamic therapy
PS	photosensitizer
MPM	Malignant pleural mesothelioma
ROS	Reactive oxygen species
IR	Infrared
OAR	Organs at risk
CT	Computed tomography
FEM	Finite element method
DIR	Deformable image registration
